# Abundant fish protein inhibits α-synuclein amyloid formation

**DOI:** 10.1038/s41598-018-23850-0

**Published:** 2018-04-03

**Authors:** Tony Werner, Ranjeet Kumar, Istvan Horvath, Nathalie Scheers, Pernilla Wittung-Stafshede

**Affiliations:** Department of Biology and Biological Engineering, Chalmers University of Technology, 412 96 Gothenburg, Sweden

## Abstract

The most common allergen in fish, the highly-abundant protein β-parvalbumin, forms amyloid structures as a way to avoid gastrointestinal degradation and transit to the blood. In humans, the same amyloid structures are mostly associated with neurodegenerative disorders such as Alzheimer’s and Parkinson’s. We here assessed a putative connection between these amyloids using recombinant Atlantic cod β-parvalbumin and the key amyloidogenic protein in Parkinson’s disease, α-synuclein. Using a set of *in vitro* biophysical methods, we discovered that β-parvalbumin readily inhibits amyloid formation of α-synuclein. The underlying mechanism was found to involve α-synuclein binding to the surface of β-parvalbumin amyloid fibers. In addition to being a new amyloid inhibition mechanism, the data suggest that health benefits of fish may be explained in part by cross-reaction of β-parvalbumin with human amyloidogenic proteins.

## Introduction

A unifying molecular event in neurodegenerative disorders is aberrant self-assembly of proteins into amyloid fibers with a hallmark cross-β-sheet arrangement. Parkinson’s disease (PD) is the second most common neurodegenerative disorder (after Alzheimer’s disease) and the most common movement disorder. PD is characterized by widespread deterioration of subcortical structures of the brain, especially dopaminergic neurons in the substantia nigra^1^. Conformational changes resulting in assembly of the intrinsically-unstructured protein α-synuclein (αS) into amyloid fibers is directly related to PD^2,3^. The exact function of αS is unknown, but it is suggested to be involved in synaptic-vesicle release and trafficking, regulation of enzymes and transporters, and control of the neuronal apoptotic response^4,5^. αS is present at presynaptic nerve terminals^6–8^ and, intriguingly, also in many cells outside the brain. Of importance for initiation and spreading of PD, it was shown recently that αS is expressed in enteroendocrine cells of the gut epithelium; these cells directly connect to αS-containing nerves and thus form a neural circuit from the gut to the brain^9^.

αS assembles via oligomeric intermediates to amyloid fibrils under pathological conditions^10^. Although soluble αS oligomers have been proposed to be toxic^11,12^, work with pre-formed αS fibrils have demonstrated that the amyloid fibrils themselves are toxic and can be transmitted from cell to cell and are also able to cross the blood-brain barrier^13–15^. Many synthetic as well as naturally-occurring small molecules can modulate αS amyloid formation *in vitro* and cross-reactivity with other amyloidogenic proteins have been demonstrated, e.g., amyloid-β in Alzheimer’s and amylin in type-2 diabetes^16^. It was recently speculated that the gut microbiome can modulate PD progression^17^ and bacterial proteins affect αS amyloid formation *in vitro*^18^.

Despite the initial association of amyloids with proteins involved in neurodegenerative disorders, an increasing number of proteins from all kingdoms of life have been reported to form functional as well as pathological amyloids^19^. For example, biofilms are structures used by bacteria to adhere to surfaces which contain amyloids in the form of curli^20,21^. In humans, amyloids of the protein Pmel17 template and accelerate covalent polymerization of reactive small molecules into the pigment melanin and the factor XII protein of the hemostatic system is activated by amyloid formation^22^. Recently, it was revealed that food allergens may adopt amyloid states in order to survive the harsh conditions during the gastrointestinal transit. This phenomenon has been reported for allergenic proteins in various food, such as β-lactoglobulin, caseins, ovalbumin, lysozyme, and β-parvalbumin^23^. For β-parvalbumin, it was deduced that the low pH in the gut triggered calcium ion release and the resulting apo-protein then assembled into amyloids. Moreover, an amyloidogenic state of β-parvalbumin was necessary for its ability to bind immunoglobulin E (IgE) and trigger hypersensitivity in the host^23,24^. Thus, the amyloid state may play a distinct function in epitope presentation of proteins causing allergies.

Fish β-parvalbumins represent the major allergen in fish hypersensitive patients and are small, calcium-binding proteins with three EF-hand motifs of which one is non-functional^20,21,25^. Most fish species are rich in β-parvalbumins with about 0.2 g of such protein per 100 g muscle tissue^26^. This protein has been evaluated as a compliance marker for fish intake in human diet interventions and epidemiological studies since humans express mostly another isoform, α-parvalbumin^27^. Despite triggering allergies in a fraction of the population, fish is considered beneficial against several age-related diseases such as cardiovascular disease^28,29^ as well as dementia and Alzheimer’s disease^30^. Favorable effects are popularly ascribed to omega-3 fatty acids^31^, but direct evidence is lacking^32^ and thus other fish components may as well be responsible.

Because human amyloidogenic proteins can cross-react, one may speculate that fish β-parvalbumin may have ability to interact with human amyloidogenic proteins. Because the protein is highly abundant in fish and transverse to the host blood upon eating fish, this becomes a relevant question. To test this hypothesis, we here probed the putative cross-talk between Atlantic cod β-parvalbumin (Gad m 1), here abbreviated PV, and human αS using a battery of biophysical methods. This particular β-parvalbumin was previously shown to adopt a highly-stable amyloid state in the absence of calcium ions (Ca) that was recognized by human serum IgE more strongly than the monomer^23,24^. Our cross-reactivity experiments *in vitro* presented here demonstrate that PV amyloids block αS amyloid formation via a mechanism that involves binding of αS monomers to the PV amyloids. Thus, we speculate that fish intake may provide health benefits through PV amyloid interactions that prevent neurodegenerative processes.

## Results

**Figure 1:**
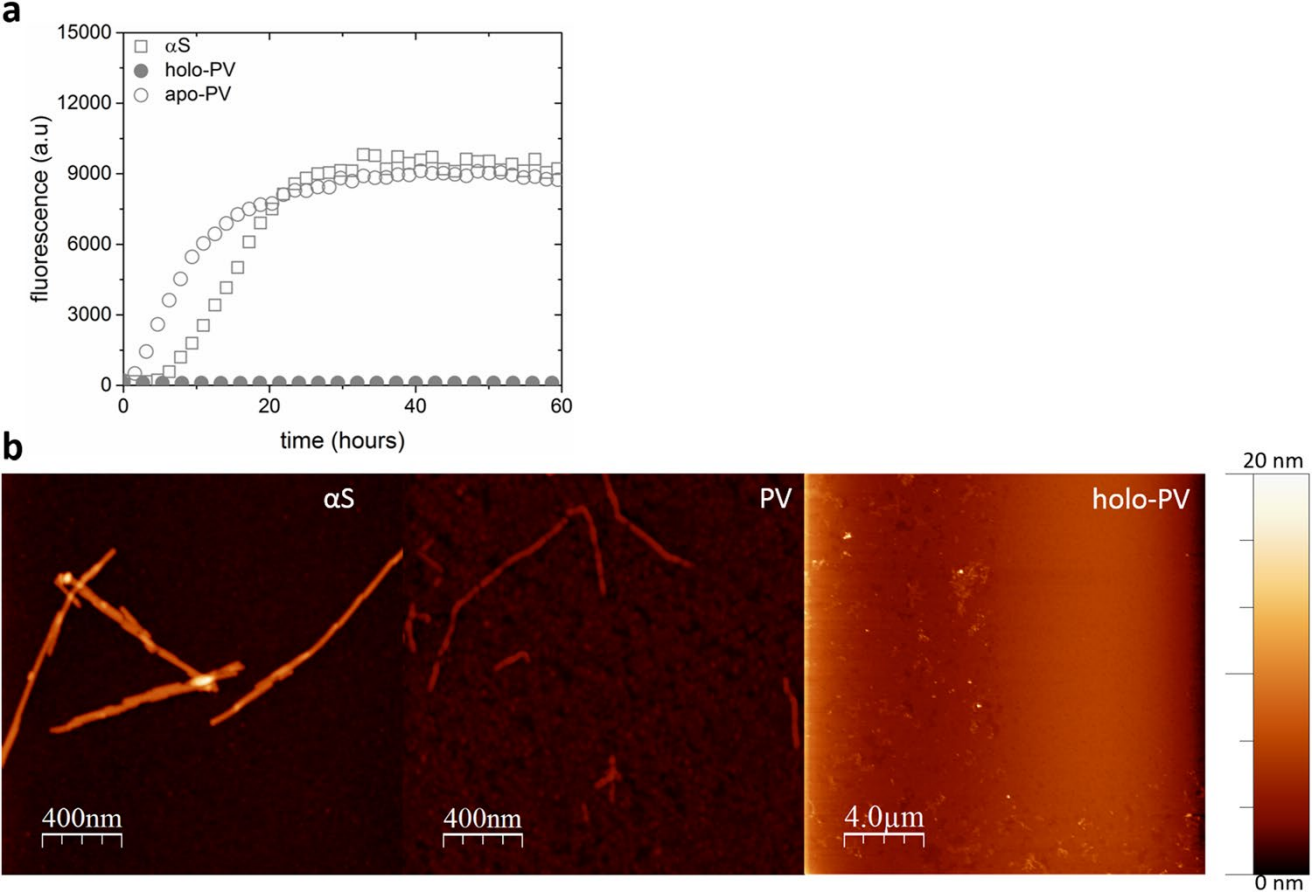
Amyloid formation of αS and apo/holo PV individually. (**a**) ThT fluorescence as a function of time for 70 µM αS, 280 µM apo- and holo-PV as individual samples. Representative curves shown (for all curves see SI). (**b**) AFM images of the end point samples (60 h) after ThT experiments for αS, apo-PV, and holo-PV with legend relating height to color.

Purified PV (Figure S1A) exhibited a far-UV CD spectrum as expected for a folded, mostly-helical protein and, as described previously, upon Ca addition, the negative CD signal was increased due to ordering of one EF hand (Figure S1B). Next, thioflavin T (ThT) fluorescence was used to monitor amyloid formation, as commonly used in *in vitro* amyloid experiments^33,34^. As also reported previously^23,24^, PV in the absence of Ca (apo-PV) is aggregation prone and forms amyloids within a few hours at the conditions here (pH 7.4, 37 °C), Fig. 1a. In Figure S1C, we show the concentration-dependence of apo-PV amyloid formation and it is clear that the higher the apo-PV concentration, the shorter is the lag time before amyloids appear. Nonetheless, based on the amplitudes of the maximal ThT signals, there is a linear dependence between apo-PV concentration and amount of amyloids formed. Thus apo-PV readily forms amyloids at concentrations down to at least 35 μM (Figure S1C).

**Figure 2:**
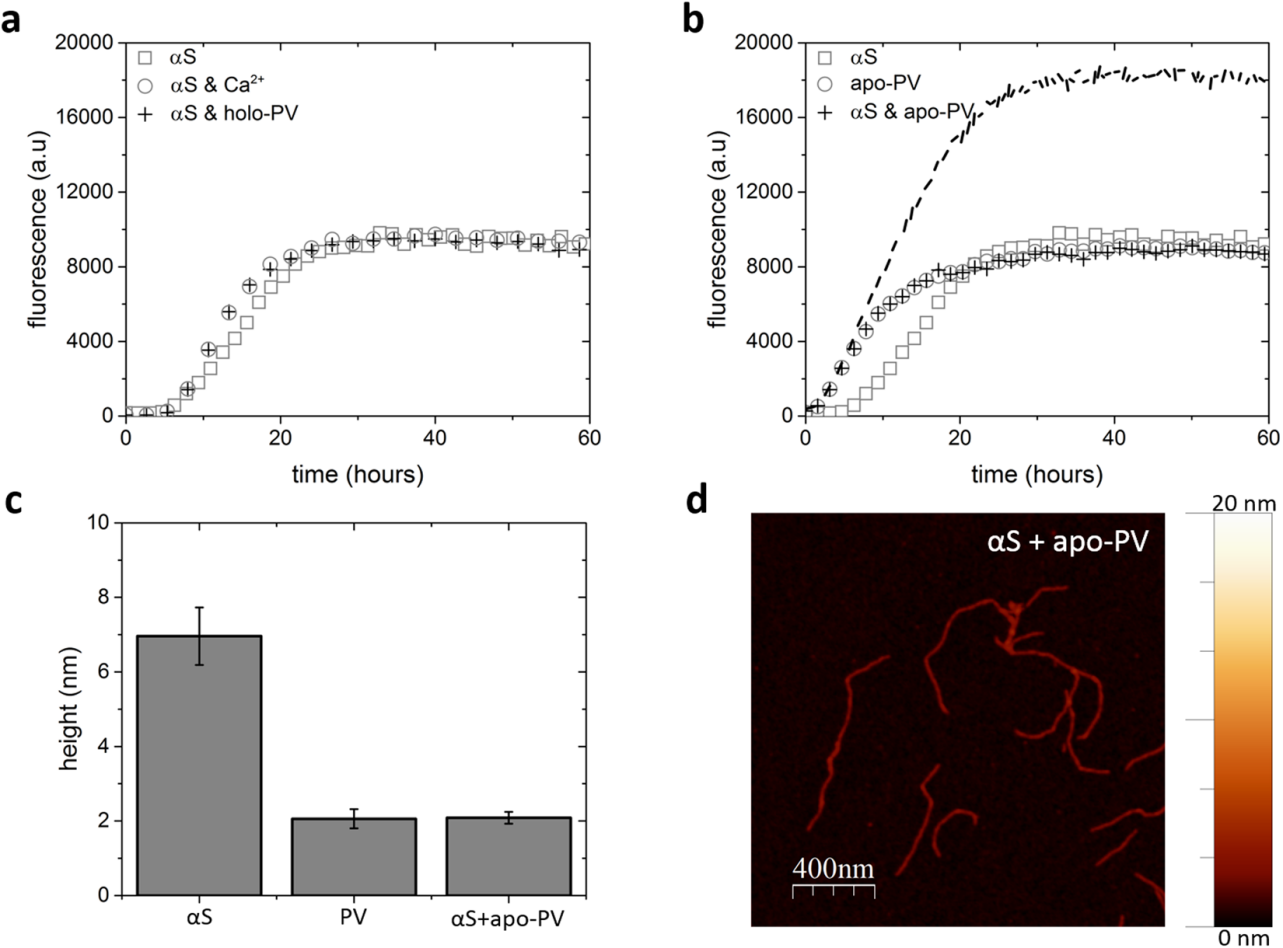
Amyloid formation of mixtures of αS and apo/holo PV. (**a**) ThT fluorescence as a function of time for 70 µM αS (with and without Ca) and αS + 280 µM holo-PV. (**b**) ThT fluorescence as a function of time for 70 µM αS, 280 µM apo-PV, and mixture of αS with apo-PV. The expected signal for the αS and apo-PV mixture, if both proteins aggregated independently, are shown as a dashed curve. (**c**) Amyloid fiber height analysis (based on 15 different fibers in each case) in the αS/apo-PV mixture, αS alone and apo-PV alone samples. (**d**) AFM image of end point in ThT experiments for αS/apo-PV mixture (more views in Figure S2B).

The resulting apo-PV amyloid fibers were analyzed with AFM and found to have fiber heights of approximately 4 nm (Figs 1b and 2c). For PV in the presence of Ca (1 mM; Ca-PV), no ThT increase was found and, in accord, no amyloid fibers were detected for Ca-PV samples by AFM (Fig. 1a,b). At the same conditions, αS also forms amyloids but with a longer lag time (Fig. 1a). The presence or absence of 1 mM Ca had no significant effect on αS amyloid formation in our experiments (Fig. 2a), although we note that Ca was reported to interact weakly with αS and affect its functions *in vivo*^35^. End-point αS amyloids are thicker (heights of around 6–8 nm in AFM) than apo-PV amyloids (Figs 1b and 2c).

To test for cross-reactivity, apo/Ca-loaded PV were mixed with αS and aggregation experiments performed. Here, 280 μM PV was selected, as that was a concentration at which PV amyloids formed faster than the aggregation process of αS (Figure S1C, Fig. 1a). We found that whereas the presence of holo-PV had no effect on αS amyloid formation (Fig. 2a), mixing of apo-PV and αS resulted in a ThT fluorescence curve exactly like the apo-PV alone one. Thus, the increased ThT emission expected upon αS amyloid formation was lacking, implying that αS amyloid formation was blocked (Fig. 2b). In accord, ultracentrifugation experiments followed by SDS-PAGE analysis of the resulting soluble fraction demonstrated that whereas fresh αS is found in the soluble fraction and aggregated αS is in the insoluble fraction, αS mixed with apo-PV stayed in the soluble fraction also after an aggregation experiment (Figure S2A). Because the kinetics of apo-PV amyloid formation was not altered in the presence of αS, inhibition of αS aggregation appeared mediated by the end product of the apo-PV reaction, *i.e*., the amyloids. AFM analysis of the resulting amyloid fibers in apo-PV/αS mixtures demonstrated that amyloid fiber dimensions are homogeneous and match those of apo-PV (Fig. 2c,d; Figure S2B). This result supports that αS aggregation is inhibited in the mixture. In support of an inhibitory process mediated by apo-PV amyloids, addition of pre-formed PV amyloid fibers to fresh αS samples, also resulted in inhibition of αS amyloid formation (Figure S2C). Therefore, the total concentration of PV monomers (which exceeded biological relevance in this experiment) does not matter: instead, it is the presence (and concentration) of apo-PV amyloids that determines the inhibitory effect on αS amyloid formation. Notably, in Figure S1C, we demonstrate apo-PV amyloid formation at concentrations down to 35 μM, which is within the biologically-relevant range.

**Figure 3:**
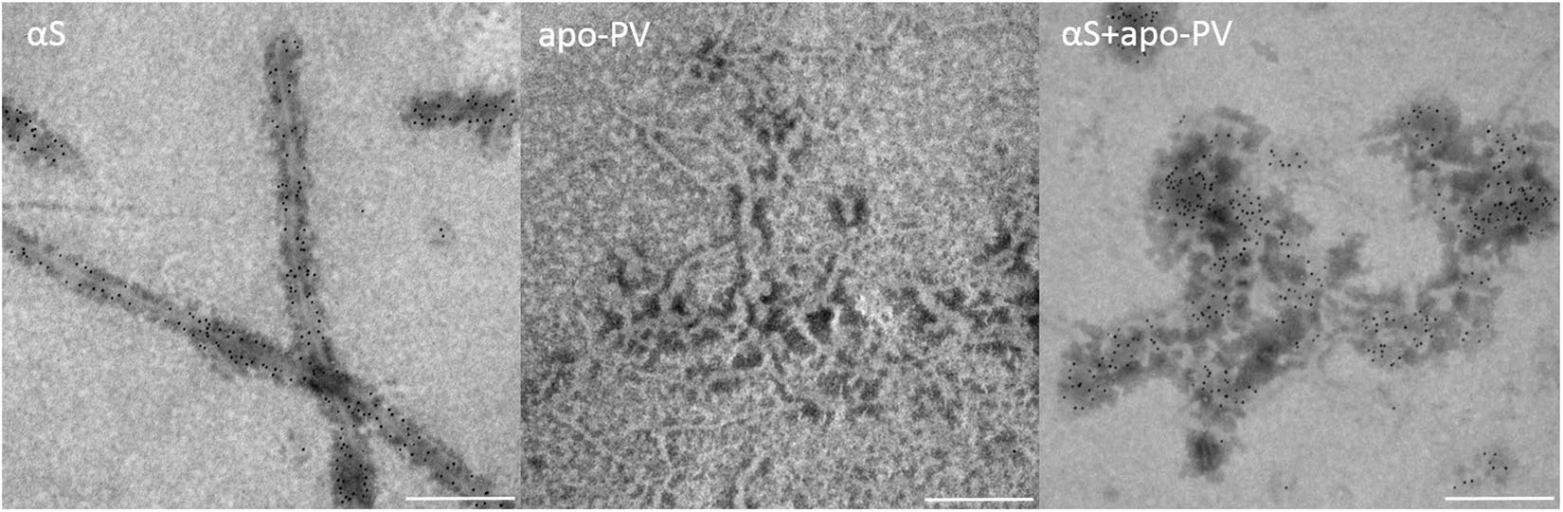
Binding of αS to apo-PV amyloid fibers. Pre-formed amyloids of αS alone, apo-PV alone and αS/apo-PV mixture were investigated by TEM upon the addition of anti-αS antibodies (monoclonal antibody; not conformation specific) coupled to gold nanoparticles (AuNP) adsorbed to secondary antibodies (scale bar: 200 nm).

To directly test if inhibition of αS amyloid formation is due to αS binding to the surface of PV amyloids, we used nanoparticles coupled to 2° antibodies to αS-reactive antibodies. TEM analysis of incubated apo-PV/αS mixtures clearly shows that αS is present at the PV amyloids, but no nanoparticles are detected when PV-alone amyloid samples are analyzed (Fig. 3).

**Figure 4:**
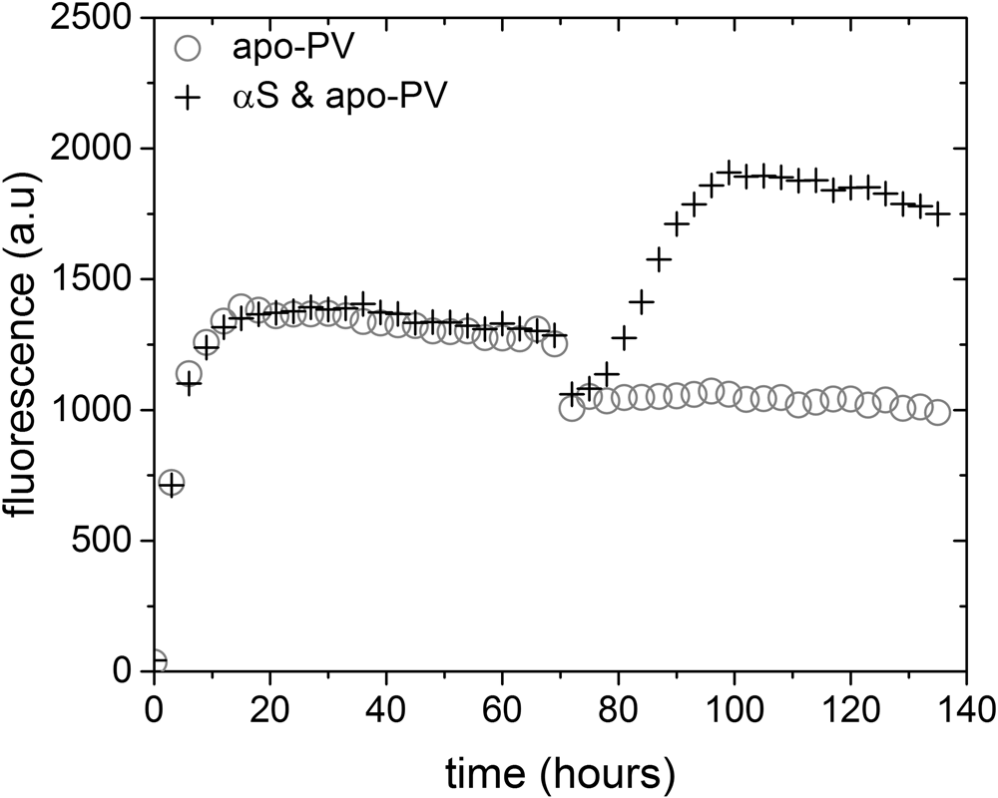
Calcium-induced release of αS from PV amyloids. 1 mM CaCl_2_ was added to ThT experiments of αS/apo-PV mixtures (and apo-PV alone as control) at time point 70 h, which is a condition when apo-PV has formed amyloids and αS aggregation remains blocked. The instant drop in signal for both curves at the 70 h time point is due to mixing.

Analysis of the PV sequence has revealed what peptide segments are in the amyloid core^23,24^. The two Ca-binding loops are not part of the amyloid core (Figure S3A) and we thus expected that PV amyloids could bind Ca. If Ca is added to pre-formed apo-PV amyloids, there is no effect on ThT emission, implying that the amyloids remain intact (Fig. 4). However, if Ca is added to incubated αS/apo-PV mixtures (thus containing PV amyloids with αS bound to the surface), we find an increase in ThT emission as if αS is now released and starts to aggregate (Fig. 4). The ThT transition appearing after Ca addition roughly matches that for αS alone (Figure S3B) and suggests that Ca competes with αS for binding to the PV amyloids, with the former having higher binding affinity which then releases αS from the PV fibers. A similar scenario is found when pre-formed αS amyloid seeds are added to the αS/apo-PV mixture. Although αS aggregation inhibition is noted at first, despite the addition of αS seeds, with time αS starts to aggregate; this is in accord with the αS amyloid seeds, eventually, pulling αS away from the PV amyloids (Figure S3C). Taken together, these results suggest that the inhibition resulting from αS binding to PV amyloids is a kinetic sequestering effect; the αS amyloid structure is still a more stable state than the PV-bound one.

## Discussion

Fish is commonly known as ‘healthy food’. From a scientific point of view, studies imply that diets rich in fish correlates with better health and less neurodegeneration^30,31^. But what are the underlying reasons? We here showed that an abundant protein in fish, PV, can interact with αS, the major player in PD, and block its amyloid formation *in vitro*. PV is found in all kinds of fish but it is especially abundant in cod, carp, redfish and herring^36^. Thus, in addition to omega-3 fatty acids, PV from fish may be responsible for favorable health effects with respect to age-related dementia and cognition decline.

We determined that PV amyloids inhibit αS amyloid formation by scavenging αS monomers to the PV amyloid fiber surface, possibly by interactions with protruding Ca-binding loops. This (i.e., binding of an amyloidogenic protein to the surface of preformed amyloids of another protein, with the latter acting as the inhibitor) is an inhibitory mechanism not described before, but it is somewhat similar to colloidal inhibition where aggregates of small molecules/proteins accumulate the amyloidogenic protein non-specifically. Since αS binding to PV amyloid fibers did not affect PV amyloid formation kinetics per se, one may speculate that secondary nucleation and fragmentation are not important in the intrinsic PV amyloid formation process. However, these processes may not necessarily be affected by αS binding. Instead, the finding that PV amyloid seeds do not speed up PV amyloid formation (Figure S4), provides the best support against a role of secondary nucleation in the PV amyloid formation mechanism. Amyloid formation of PV appears thus dictated by the product of the rates of elongation and primary nucleation processes^37^.

In contrast to what we find for PV and αS here, the structurally-related protein calmodulin was found to interact with αS only in its Ca-bound state^38^. For the latter complex, the positively-charged N-terminus of αS was responsible for interaction with calmodulin^38^; thus, one may speculate that the αS N-terminus is involved also in the interaction with the apo-PV amyloids.

Because of the overwhelming increase in amyloid disorders (e.g., Alzheimer’s disease, PD, and type-2 diabetes) predicted for the world’s population in the near future, and the current lack of medical cures^39^ (only symptomatic drugs exist), all new approaches that may curb amyloid formation are of high interest^40^. After a meal of fish, PV can be found in the blood^27^ (and this triggers an immune response in some people) and, at least in the gut, before reaching the blood, PV is in an amyloid form^23,24^. It is not clear where PD starts but it is proposed that it may originate from the gut via the enteric nerve system^9,17^.Based on our i*n vitro* results of inhibition of αS amyloid formation, which clearly must be followed by many *in vivo* studies, we speculate that eating PV-rich fish is a dietary recommendation that may prevent or delay PD.

## Materials and Methods

### Protein expression and purification

The human αS construct was transformed into BL21 (DE3) (Novagen) cells. Transformants were first grown to an OD_600_ of 0.6 in LB containing 100 µg/ml carbenicillin at 37 °C, then induced with 1 mM isopropyl b-D-1-thiogalactopyranoside(IPTG) and grown overnight at 25 °C. Cells on ice were lysed by sonication in pulse mode in 20 mM Tris-HCl buffer pH 8.0 in the presence of protease inhibitor cocktail (ref: 05892791001, Roche). After sonication, the lysate was treated with a universal nuclease (Pierce) for 15 min at room temperature. The lysate was then heated at 90 °C for 10 min followed by centrifugation for 30 min at 15 000 g. The centrifuged lysate after filtration (Nalgene rapid-flow filter, 0.2 µm PES membrane; Thermo Fisher Scientific) was loaded on a pre-equilibrated 5 mL HiTrap Q FF anion exchange column (GE Healthcare) and eluted by a linear gradient of 1 M NaCl in 20 mM Tris-HCl, pH 8.0. Fractions contained αS were combined and concentrated with Ultra-15 Ultracel 10 K centrifugal filter devices (Millipore). The concentrate was loaded on to HiLoad 16/600 Superdex 75 pg column (GE Healthcare) and retrieved in 20 mM Tris-sulfate buffer, pH 7.4. Purity was confirmed by a single-band on SDS-PAGE gel and a single elution peak in SEC. Protein samples were flash frozen and stored at −80 °C until use. To determine protein concentrations, the extinction coefficient of 5700 cm^−1^ M^−1^ at 276 nm for αS was used.

The Atlantic cod β-parvalbumin (A51874, Gad m1; here termed PV) gene in a pET15b vector was transformed into BL21 (DE3) competent cells and grown in LB medium containing 100 mg/L carbenicillin at 37 °C until an OD_600_ of ~0.6. Protein expression was induced by IPTG and incubated overnight at 27 °C. The cells were centrifuged at 4100 × g for 20 min, supernatant decanted and pellet re-suspended in 10 mM Tris-HCl with a protease inhibitor cocktail (ref: 05892791001, Roche), pH 7.8, sonicated on ice followed by centrifugation at 13 200 × g for 30 min at 4 °C. Supernatant was recovered and diluted two times with 40 mM Tris-HCl, 0.2 M NaCl, 10 mM imidazole, pH 7.8, 50 µM CaCl_2_ and cleared by repeating the centrifugation step followed by filtration (Nalgene rapid-flow filter, 0.2 µm aPES membrane, Thermo Fisher Scientific). Hisprep FF 16/10 (GE Healthcare) equilibrated with 20 mM Tris-HCl, 100 mM NaCl, 5 mM imidazole, pH 7.8 and gradient eluted with increasing strength of the same buffer but with 500 mM imidazole. Finally, the collected eluate was run through size exclusion chromatography (HiLoad 16/600 Superdex 75 pg, GE Healthcare) equilibrated with 25 mM Tris-HCl and 1 mM CaCl_2_. Protein purity was assessed by SDS-PAGE (Figure S1A) and fractions containing PV were flash frozen and stored at −80 °C. PV concentrations were determined by the extinction coefficient of 1950 cm^−1^ M^−1^at 257 nm. In order to remove Ca from holo-PV, i.e., to create apo-PV, 5 mM EDTA was included in experiments involving the apo-PV form.

### AFM

End-products of ThT experiments were diluted 10–20 times in milli-Q water and incubated on freshly cleaved mica for 10 min, after which the mica was rinsed with milli-Q water and dried under a nitrogen stream. NTEGRA Prima setup (NT-MDT) was used in conjunction with gold-coated single crystal silicon cantilever (NSG01, spring constant of ~5.1 N/m; NT-MDT) at a resonance frequency of ~180 kHz. A 0.5 Hz scan rate was used to acquire the 512 pixel images that were subsequently analyzed by the use of WSxM 5.0 software^41^.

### Thioflavin T (ThT) assay

Immediately prior to aggregation experiments, thawed αS was purified by SEC to remove oligomeric species; the monomer fraction was collected and used as the αS starting material in aggregation experiments^42^. Amyloid formation experiments of αS and apo- and holo-PV were performed during agitation in a plate reader (Fluostar Optima or Fluostar Omega; BMG Labtech) in 25 mM Tris-HCl, 0.15 M NaCl, pH 7.4 together with 20 µM recrystallized ThT (T3516; Sigma-Aldrich) at 37 °C. A 2 mm glass bead was present in all samples. An EDTA concentration of 50 µM was used in holo-PV experiments, and 5 mM EDTA was used in apo-PV experiments. Samples were excited at 440 nm and ThT fluorescence recorded at 480 nm every 20 min. The ThT experiments were performed in at least triplicates and representative curves were chosen for the presented figures. The reproducibility was high (see Figure S5 for all replicates of individual ThT experiments).

### TEM

A 10 µl volume of aggregated protein was incubated for 10 minutes on Formvar-coated 200 mesh size copper grids and then blocked with PBS-BSA (0.01 M Na_2_PO4, 0.0027 M KCl, 0.137 M NaCl, 1% BSA, pH 7.4) (Sigma-Aldrich) for 10 min. The grid was then incubated with a mouse monoclonal antibody targeting αS (Syn211, Thermo Fisher Scientific; not specific to αS conformation or assembly status), diluted 1:5 in PBS with 1% BSA, for 30 min. The sample was then washed with PBS containing 0.1% BSA three times 5 min each followed by an incubation with donkey anti-mouse antibody with pre-adsorbed 6 nm gold particles (ab105276, Abcam), 1:5 dilution in PBS (1% BSA) for 30 min. The washing step was repeated followed by cross-linking with 1% glutaraldehyde for 10 min. Finally, the grid was washed with milli-Q, 3×5 min and negatively-stained with 1% phosphotungstic acid hydrate (79690, Sigma-Aldrich) for 30 s.

### Data availability statement

All data generated and analyzed during this study are included in this published article and its Supplementary Information.

## Electronic supplementary material

supplementary information
