# Supplementary material for: Abundant fish protein inhibits α-synuclein amyloid formation
Source: Sci Rep. 2018 Apr 3;8:5465. doi: 10.1038/s41598-018-23850-0 (PMC5882657; doi:10.1038/s41598-018-23850-0)
Supplement: Supplementary file 1 — supplementary information [file 41598_2018_23850_MOESM1_ESM.pdf]

Supporting Information

Abundant fish protein inhibits α-synuclein amyloid formation

Tony Werner, Ranjeet Kumar, Istvan Horvath, Nathalie Scheers, and

Pernilla Wittung-Stafshede

Content:

Figures S1-S5

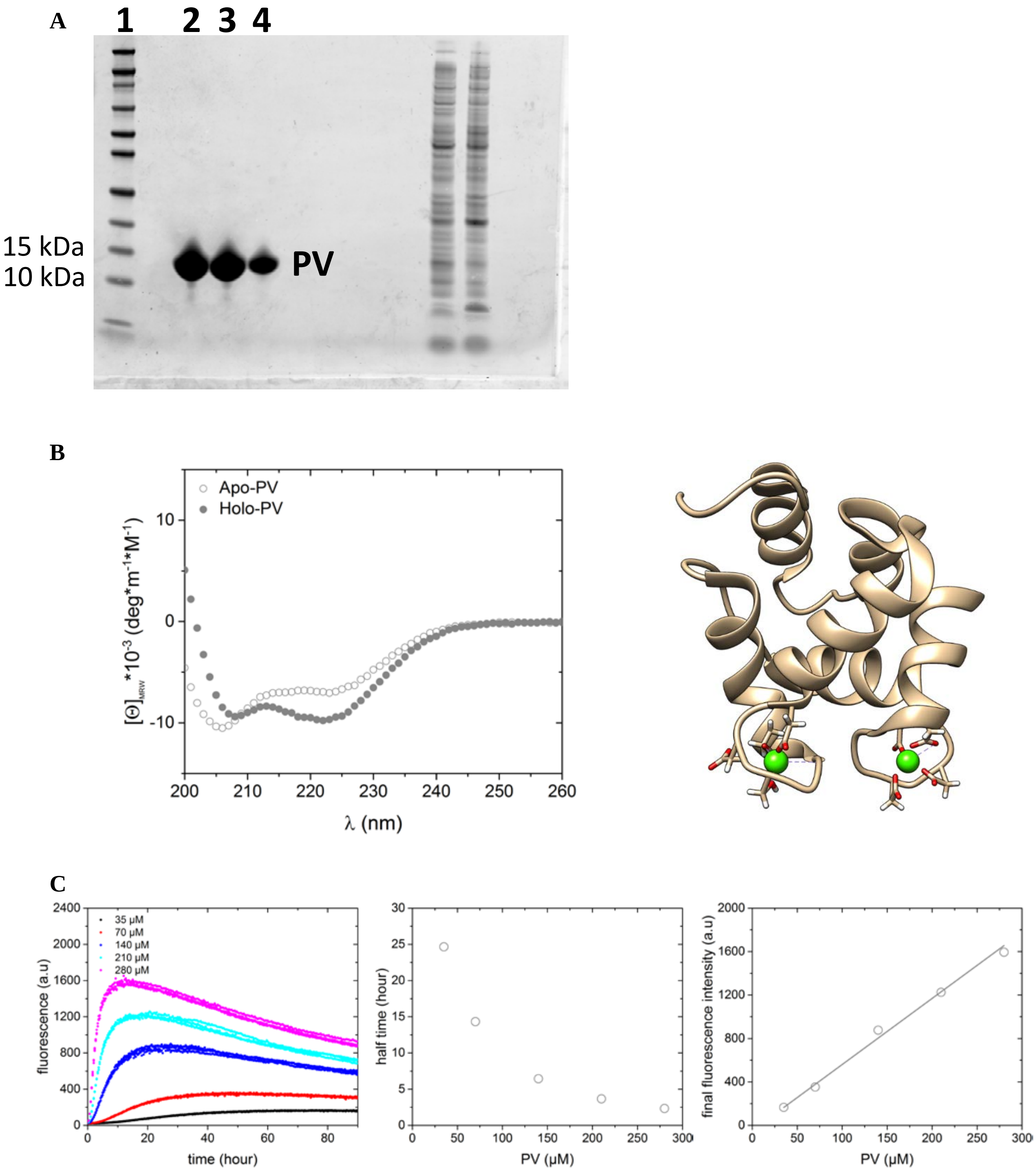

Figure S1

A. SDS PAGE of purified PV (lanes 2-4) along with molecular weight markers (lane 1). The two rightmost lanes are from an independent experiment. Image contrast increased (auto function) using ImageJ (ver 1.6.0_24, NIH). B. *Left*. Far-UV CD of apo-PV and holo-PV indicating structural ordering upon Ca binding, as previously reported (DOI: 10.4414/smw.2015.14128). *Right*. Structural model of holo-PV based on a high resolution NMR structure (PDB ID: 2MBX) with Ca ions shown as green spheres and coordinating ligands (CD loop: Asp52, Asp54, Ser56, Glu63; EF loop: Asp91, Asp93, Asp95, Glu102) highlighted in stick representation. C. *Left*. Concentration dependence of apo-PV amyloid formation as probed by the ThT assay (triplicates). *Middle*. Half time of amyloid formation (time to reach half-maximal ThT signal) plotted against apo-PV concentration. *Right*. Maximal ThT fluorescence (at highest point) versus apo-PV concentration (R^2: 0.9957).

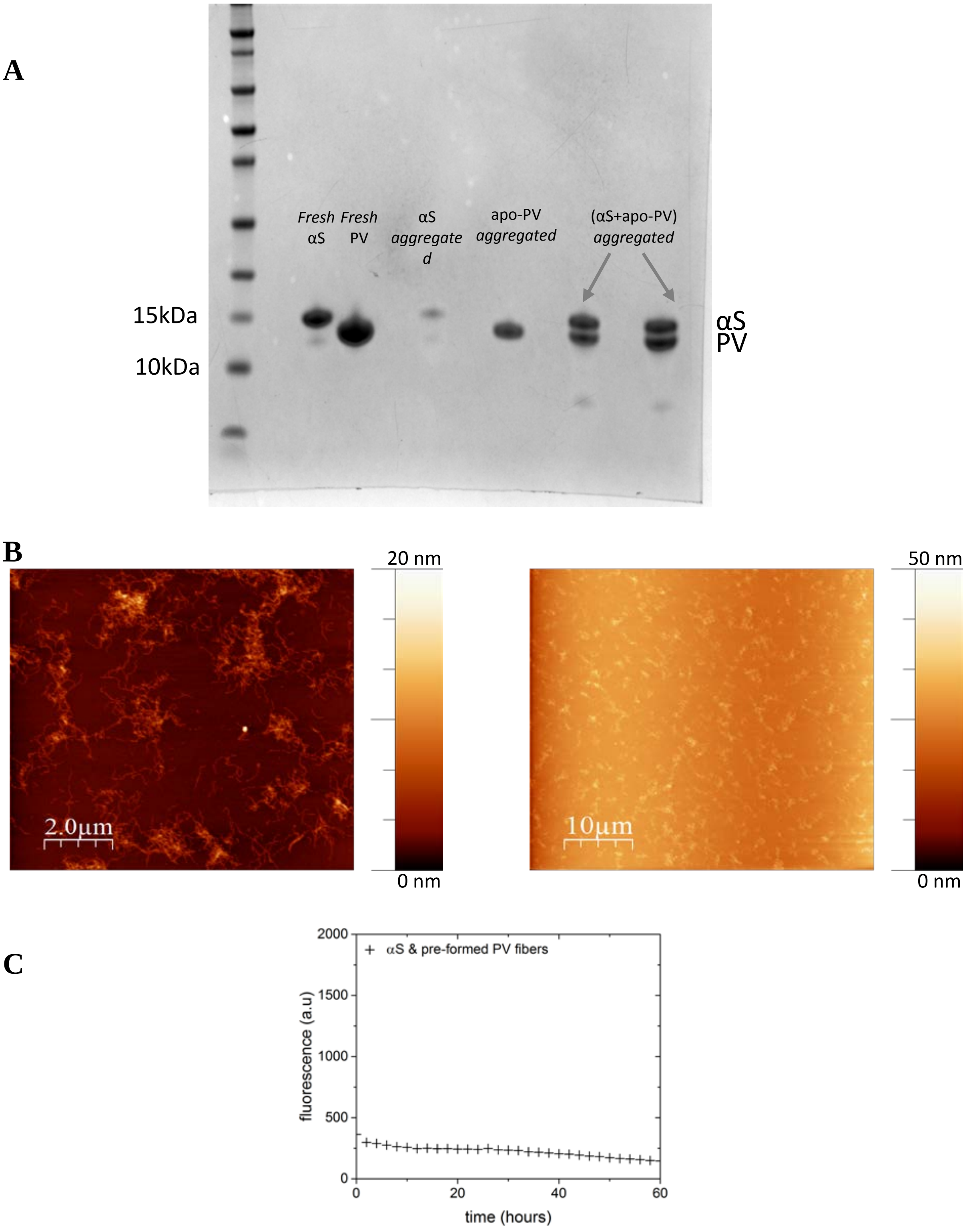

Figure S2

A. SDS-Page analysis of samples after ultracentrifugation of fresh αS, aggregated αS, and aggregated mixture of αS+apo-PV (two samples of the latter). In individually-aggregated αS and apo-PV samples, less protein is found in the soluble fraction as compared to for fresh samples. When αS is aggregated with apo-PV, more αS is found in the soluble fraction when compared to αS aggregated alone, in accord with PV-mediated inhibition of αS-amyloid formation. B. AFM images with 2 μm and 10 μm field of views of endpoint samples from aggregation experiments of αS/apo-PV mixtures (also shown in Fig 2D). C. ThT assay for αS aggregation upon the addition of pre-formed apo-PV amyloid fibers (280 μM) at time zero. The data imply that pre-formed apo-PV amyloids block αS aggregation.

A

amyloid core *Ca^{2+}-specific site*

MAFAGILNDADITAALAACKAEGSFDHKAFFTKVGLAAKSSADIKKVFEIIDQDKSDFVEEDE
LKLFLQNFSAGARALSDAETKVFLKAGDSDGDGKIGVDEFGAMIKA

amyloid core *Ca^{2+}/Mg^{+2} site*

B

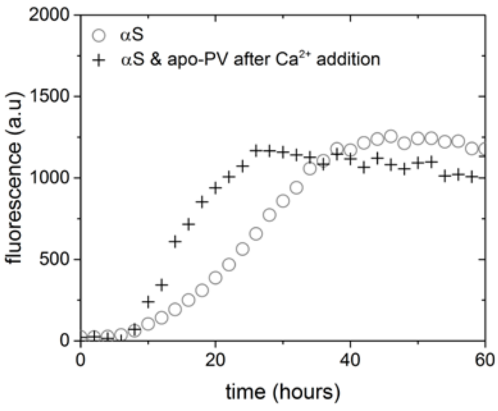

C

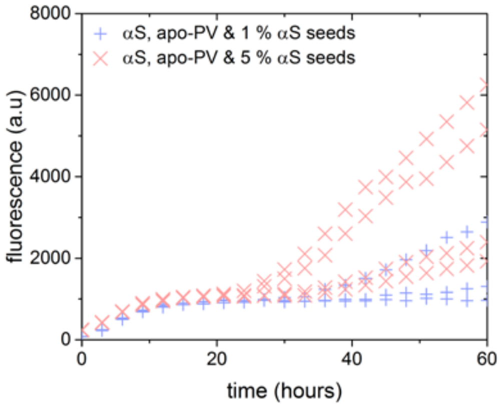

Figure S3

A. Amino acid sequence of PV with amyloid core regions (red) and metal (Ca/Mg) sites (grey) indicated. The data shows that amyloid stretches and Ca^{2+} and Ca^{2+}/Mg^{2+} binding sites do not overlap (doi:10.1038/srep32801). B. Comparison of ThT fluorescence kinetics for αS alone (data in Figure 1A), and when starting from Ca addition to a αS/apo-PV pre-aggregated mixture (data in Figure 2A; time point 70 h set to zero). C. ThT assay for αS/apo-PV mixture with the addition of pre-formed αS amyloid seeds (1 %, blue, 3 individual traces; and 5 %, red, 4 individual traces) at the start. The data initially match that in Figure 2A, for αS/apo-PV mixture alone, but at longer time scales (> 30 hours), the inhibitory effect is lost and αS appears to begin to aggregate.

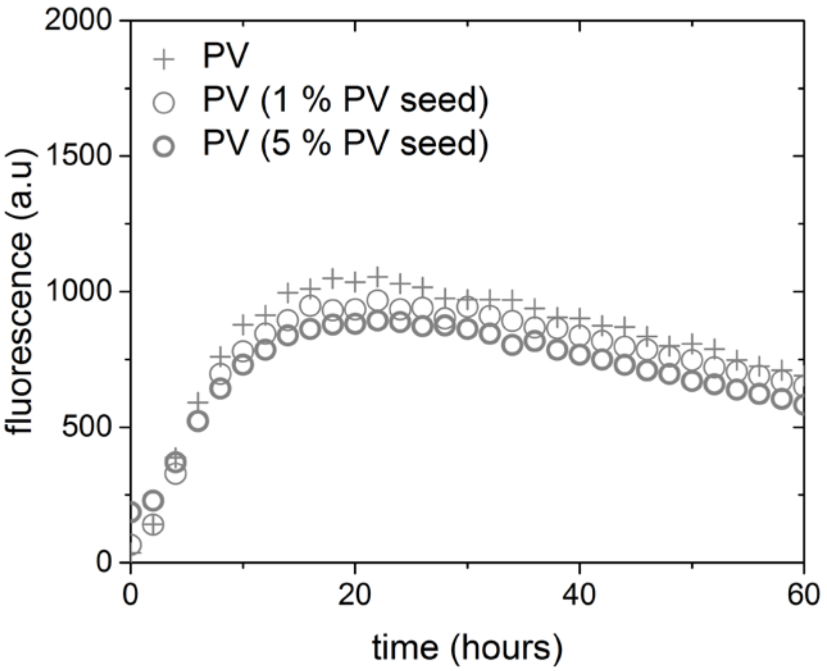

Figure S4
ThT experiments probing seeding of apo-PV amyloid formation by pre-formed PV amyloids PV seeds (1 % and 5 %). No effects were found by seeds, indicating that other processes than nucleation to dominate the kinetics of PV amyloid formation.

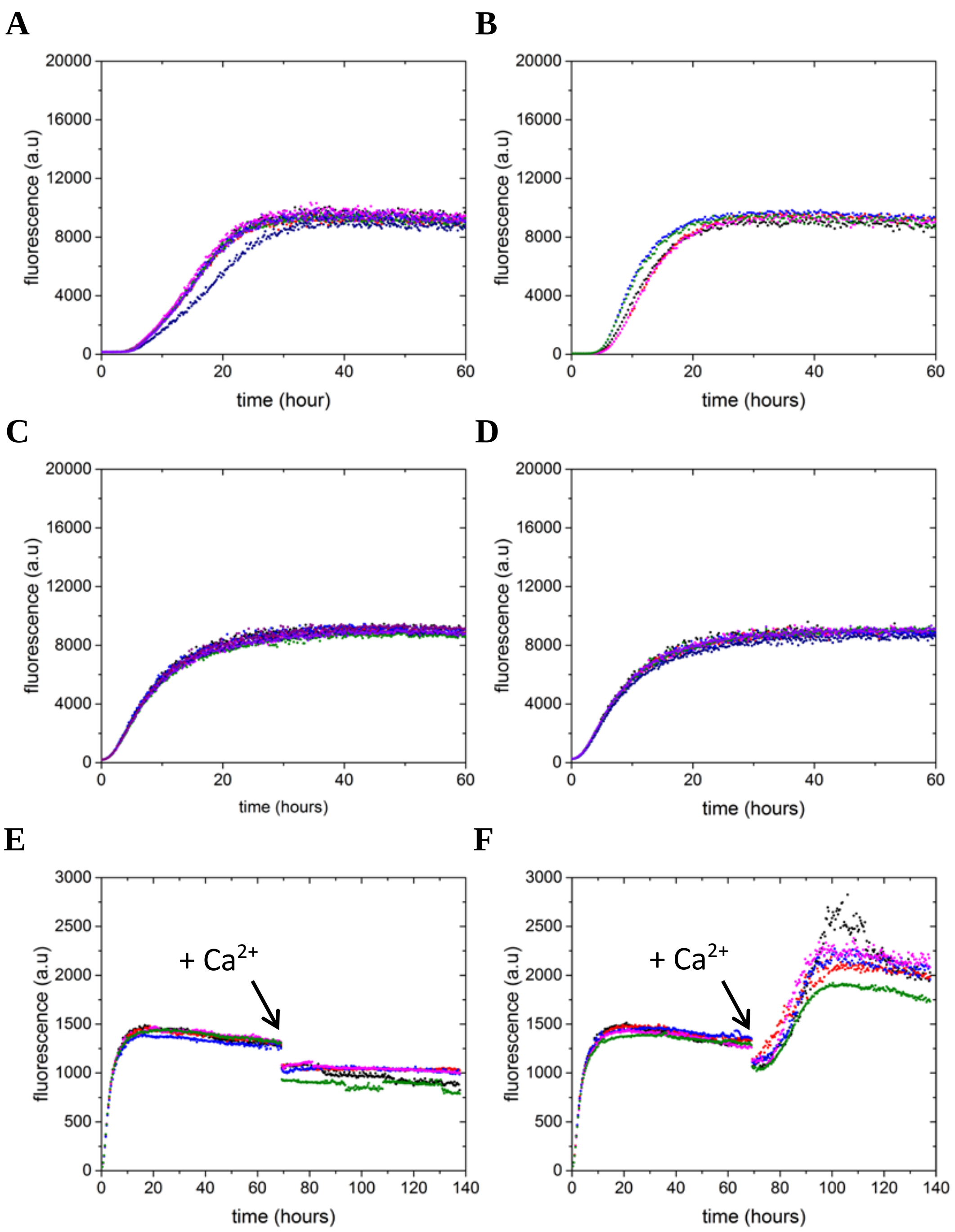

Figure S5
Replicas of experiments show good reproducibility. ThT data (individual traces) for aggregation of 70 μM αS (seven replicates) (A), 70 μM αS with 1 mM Ca (five replicates) (B), 280 μM apo-PV (eight replicates) (C), 70 μM αS mixed with 280 μM apo-PV (seven replicates) (D), 280 μM apo-PV with 1 mM Ca addition at time point 70 h (five replicates) (E), and finally, 70 μM αS mixed with 280 μM apo-PV with 1 mM Ca addition at time point 70 h (five replicates) (F).
